# Assessment of cutaneous involvements by F-18 fluorodeoxyglucose positron emission tomography imaging in patients with adult T-cell leukemia/ lymphoma

**DOI:** 10.1186/1742-4690-8-S1-A52

**Published:** 2011-06-06

**Authors:** Masahiro Amano, Mitsuru Setoyama

**Affiliations:** 1 Department of Dermatology, University of Miyazaki Faculty of Medicine, Miyazaki, Japan

## 

F-18 fluorodeoxyglucose positron emission tomography (FDG-PET) imaging has been shown to be useful in the evaluation of many tumors due to its high sensitivity and specificity. FDG-PET examination is also useful in Hodgkin and B-cell lymphomas. Few data have been reported on T-cell, natural killer (NK)-cell lymphomas and adult T-cell leukemia/lymphoma (ATLL). A retrospective review of patients with ATLL who underwent FDG-PET examination at our institute for initial disease staging from 2008 to 2010 was undertaken. FDG-PET data were compared with histopathological findings of cutaneous involvements of ATLL. ATLL subtypes were diagnosed according to Shimoyama’s diagnostic criteria and our criteria for cutaneous ATLL. Fifteen patients with ATLL were included in this study. There were 8 men and seven women, at a median age of 66.7 (51 – 91) years. The diagnoses included cutaneous type ATLL (n=13) and chronic type ATLL (n=2). All of those 15 patients had cutaneous involvements and 8 (53%) had FDG-avid cutaneous involvements. The maximum standardized uptake value (SUV) was recorded for each patient. The mean SUV of abnormal foci in cutaneous involvement of ATLL was 4.76 mg/ml (range: 1.46 – 11.4). In all cases of chronic type ATLL (n=2), cutaneous involvements were FDG-avid and in seven cases of cutaneous type ATLL (n=13), cutaneous involvement were not FDG-avid.

In conclusion, further study will be required to determine the diagnostic value of the initial FDG-PET in the patients with ATLL.

